# Serum/Plasma MicroRNAs as Biomarkers for HBV-Related Hepatocellular Carcinoma in China

**DOI:** 10.1155/2015/965185

**Published:** 2015-01-22

**Authors:** Wen Yin, Yan Zhao, Yong-Jing Ji, Li-Ping Tong, Ya Liu, Shui-Xiang He, Ai-Qin Wang

**Affiliations:** ^1^Department of Microbiology, Fourth Military Medical University, Xi'an, Shaanxi 710032, China; ^2^Department of Gastroenterology, First Affiliated Hospital of the Medical College, Xi'an Jiaotong University, Xi'an, Shaanxi 710032, China; ^3^Department of Medicine, Jinan 2nd People's Hospital, Jinan, Shandong 250001, China; ^4^Department of Out-Patient, Xijing Hospital, Fourth Military Medical University, Xi'an, Shaanxi 710032, China

## Abstract

MicroRNAs (miRNAs) are a group of small RNAs with a fundamental role in the regulation of gene expression. These RNAs have been shown to participate in various cellular and physiological processes, including cellular development, apoptosis, proliferation, and differentiation. Aberrant expression of several miRNAs was found to be involved in a large variety of neoplasms, including hepatocellular carcinoma (HCC). Previous studies have shown the existence of a large amount of stable miRNAs in human serum/plasma, which laid the foundation for studying the role of serum/plasma miRNAs in the diagnosis and prognosis of HCC. Here, we review the recent progress in research on serum miRNAs as biomarkers for HCC in Chinese patients.

## 1. Introduction

Hepatocellular carcinoma (HCC) represents the third most common cause of death from cancer worldwide, with an increasing incidence expected in the next decades [1]. The high fatality rate demonstrates the need for specific diagnostic methods and effective therapeutic strategies for HCC [2]. Currently, the diagnosis of HCC is usually based on imaging (abdominal ultrasonography, contrast-enhanced computed tomography (CT), and magnetic resonance imaging (MRI)) and laboratory analysis (serum*α*-fetoprotein (AFP) levels), and it is sometimes verified by hepatic tissue biopsy [3]. Ultrasonography can detect large lesions but not small tumors, and the diagnostic accuracy of this operator-dependent procedure varies. Advances in MRI and CT have greatly improved the diagnostic imaging of small lesions of HCC. However, these procedures are costly, and they may not be readily available in some developing countries. Currently, AFP is the main laboratory analysis performed in the clinic for the diagnosis of primary HCC; however, its sensitivity (39–65%) and specificity (76–94%) are unsatisfactory [4]. Despite the substantial effort of doctors and investigators, a large proportion of patients with HCC are still diagnosed at an advanced stage when effective treatments are lacking [5]. Hence, the development of noninvasive biomarkers with high sensitivity and specificity that can be used for large-scale clinical investigations is greatly needed.

MicroRNAs (miRNAs), which are endogenous small noncoding RNAs consisting of 20–25 nucleotides, may regulate as much as 60% of the human genome [6]. Humans express nearly 1000 miRNAs, each with the potential to bind multiple host mRNA molecules [7]. miRNA biogenesis, which is important for various cellular and physiological processes, including cellular development, apoptosis, proliferation, and differentiation, has been well characterized and consists of several steps [8, 9]. The development of various diseases may be due to alterations in any of the steps of miRNA biogenesis [10, 11]. For instance, the deregulation of miRNAs may activate oncogenes and inactivate tumor suppressor genes in human carcinogenesis.

Several miRNAs have been linked to the initiation and progression of HCC, which may present a new avenue for the study of the molecular mechanisms, diagnosis, and implementation of novel therapeutic targets in HCC [12–15]. In 2008, Chen et al. reported an exciting discovery that human serum/plasma contains a large amount of stable miRNAs and that the unique expression profile of serum miRNAs could serve as a fingerprint for various diseases [16]. Subsequently, numerous important studies have demonstrated the potential of serum/plasma miRNAs as novel noninvasive biomarkers for the diagnosis of HCC, and most of these studies have been conducted in China [17–27]. In this paper, we discuss recent evidence related to the value of serum microRNAs as biomarkers for HBV-related HCC in China.

## 2. Methods of Literature Search

**Figure 1:**
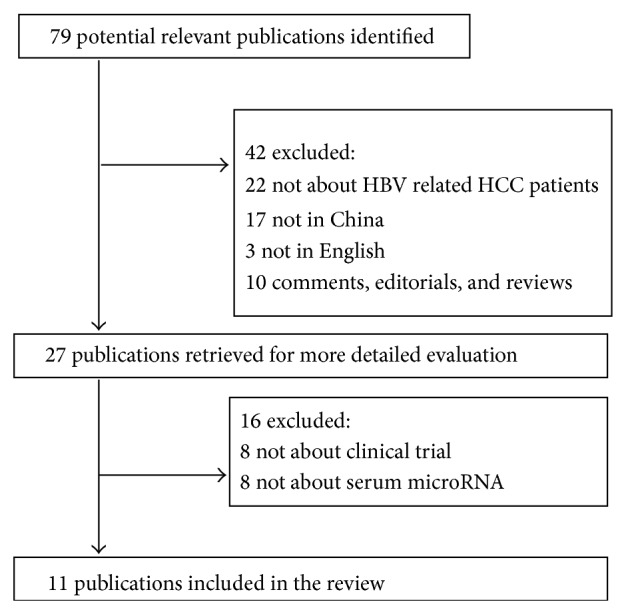
The flow chart of study section.

The following electronic databases were screened: PubMed (2005 to February 2014), PsycINFO (2005 to February 2014), Scopus (2005 to February 2014), and EMBASE (2005 to February 2014). The searches were limited to clinical trials conducted in humans. The following search terms were used: “HCC” or “liver cancer” or “liver tumor” or “hepatoma” or “hepatocellular carcinoma” AND “serum” or “plasma” AND “microRNA” or “miRNA”. The searches were limited to original articles in English, and we also screened the references of the retrieved articles. The flow chart of the systematic review is shown in Figure 1.

## 3. Result

### 3.1. Description of the Included Studies

The detailed characteristics of the included studies are described in Tables 1 and 2. Nine of the 11 trials were comparative studies. Two studies contained 3 phases: a discovery stage, a training set, and a validation set; 3 studies included 2 phases: a discovery stage and a validation set. The average age of the patients from the individual studies ranged from 37.5 to 57. In most of the trials, the blood samples of the patients were collected before any operation, chemotherapy, and/or radiation treatment was administered. Only 3 studies described the details of the inclusion criteria for the healthy control group. In 5 studies, U6 snRNAs (small nuclear RNAs) were selected as the internal normalization control; in the remaining studies, the plant miRNAs miR-168, mmu-miR-295, miR-181a and miR-181c, miR-16, and miR-1228 were used as controls. Six studies concluded that miRNAs may serve as biomarkers for the diagnosis of early-stage HCC; however, Qi et al. and Xu et al. showed that miRNAs might serve as biomarkers for liver injury but not specifically for HCC. Furthermore, the value of miRNA for providing predictive significance for the prognosis of HCC was reported in 2 other studies.

### 3.2. The Regulation of a Single Serum miRNA in HCC

#### 3.2.1. miR-122

miR-122 was identified as the most abundant liver-specific miRNA, accounting for 70% of the total hepatic miRNA [28]. Importantly, the downregulation of miR-122 was detected in more than 70% of cases of HCC [29], which may represent either a differentiation reversion or a block to a less-differentiated status of liver cells. In addition, a study by Qi et al. revealed that the level of serum miR-122 was elevated not only in HCC patients but also in HBV patients without HCC [24]. These authors' results suggested that miR-122 in the serum of patients may be a potential biomarker for liver injury [30]. Why is miR-122 downregulated in HCC tissues but elevated in the serum of HBV patients without or with HCC? Qi et al. explained that because circulating miRNAs have been shown to be stable blood-based markers [31], it is possible that miR-122 could come from damaged hepatocytes and accumulate in the blood at a high level. However, the results of the study by Qi et al. remain controversial. The data from Qi et al. indicated that the expression levels of miR-122 in the serum were significantly higher in HCC patients than in diseased controls or healthy controls, while Xu et al. showed that the expression levels of serum miR-122 were significantly higher in HBV patients than in HCC patients or healthy controls [23].

#### 3.2.2. miR-223

miR-223 (also called Mirn223) has drawn considerable attention in the literature. This myeloid-specific miRNA was reported to play critical roles in myeloid functions and differentiation [32], progenitor proliferation, and granulocyte differentiation [33]. In addition to these roles, a recent study underscored the importance of miR-223 downregulation in the development of HCC [34], suggesting a potential role of this miRNA in liver disease. Similarly, miR-122 and miR-223 were found to be downregulated in HCC tissues but elevated in the serum of HCC patients. A subsequent study demonstrated that the elevation of serum miR-223 may arise from hepatic ischemia/reperfusion injury [35]. The damage to hepatocytes in patients with chronic hepatitis may be more serious than that in patients with HCC, and it is reasonable that the level of serum miR-223 in patients with chronic B hepatitis is much higher than in patients with HCC. Qi et al. reported that the levels of miR-223 were significantly elevated in HCC patients compared with in healthy controls. Moreover, the level of serum miR-223 in HBV patients without HCC was higher than in HCC patients [24]. Hence, miR-223 may be a potential biomarker for liver diseases; however, its exact role in HCC needs to be fully investigated in the future.

#### 3.2.3. miR-21

miR-21, which is expressed in various human tissues, is another prominent miRNA implicated in the genesis and progression of human cancer. The overexpression of miR-21 has been observed in many types of cancer, such as breast cancer [36], lung cancer [37], colon cancer [38], and even HCC [15, 39]. A study by Xu et al. showed that the levels of serum miR-21 in patients with HCC was higher than that in healthy controls, which was consistent with earlier studies [23]. However, the median level of serum miR-21 in patients with chronic hepatitis was much higher than that in patients with HCC, which may suggest that the elevated serum miR-21 could also be a result of tissue injury, such as hepatitis. However, the report by Qi et al. led to further questions regarding the role of serum miR-21 in HCC. The authors found lower levels of serum miR-21 in HBV patients without or with HCC than in healthy controls, which indicates that miR-21 can act as either an oncogene or a tumor suppressor, depending on the targets that they regulate and the upstream factors that can cause the dysfunction of miR-21 [24]. Clearly, the exact role of miR-21 in cancer needs to be fully investigated in the future.

#### 3.2.4. miR-16

miR-16 was initially shown to play a potential role in chronic lymphoma leukemia [40]. However, several studies reported that it is frequently absent or downregulated in chronic lymphoma leukemia as well as in some solid tumors [41, 42]. Several miR-16 target genes have been identified by proteomic and transcription analyses, including BCL2, MCL1, CCND1, and WNT3A [27, 43, 44]. These target genes are involved in cell growth, the cell cycle, oncogenesis, tumor suppression, and apoptosis inhibition. A study by Qu et al. suggested the potential use of circulating miR-16 in the diagnosis of HCC [45]. The results of this study showed that the serum levels of miR-16 were significantly lower in patients with HCC than in chronic liver diseases patients. Furthermore, the use of miR-16 measurement as a second line of testing for cases considered negative by conventional HCC markers holds potential to improve the sensitivity [45]. However, the author's conclusion is highly controversial. In a recent study, Liu et al. did not find miR-16 to be significantly differentially expressed between tumor and adjacent normal tissues [20]. Thus, larger, prospective studies are needed to explore the role of circulating miR-16 in the diagnosis of HCC.

#### 3.2.5. miR-221

Earlier studies have shown that miR-221 is overexpressed in human tumor tissues, such as breast cancer, colorectal cancer, and glioblastoma [38, 46–49]. miR-221 was reported to stimulate the onset of tumors and promote tumor progression [13]. With the increasing focus on the study of serum miRNA, the actual role of serum miR-221 expression in HCC has been explored recently [50]. The report from Li et al. showed that individuals with HCC had significantly elevated levels of serum miR-221, and a high level of miR-221 expression was correlated with tumor size, cirrhosis, and tumor stage. Moreover, patients with high serum miR-221 levels had a significantly lower survival rate than those with low expression levels, which suggests that serum miR-221 can be used as a potential predictor of prognosis in HCC [50]. However, the study results were limited by the limited number of screened microRNAs (only four miRNAs were selected in the pilot study) and the small sample size (only 46 HCC patients were investigated).

#### 3.2.6. miR-129-2

Previous studies have shown that miR-129-2 is dysregulated and heavily methylated in many types of cancers, such as gastric cancer, endometrial cancer, esophageal squamous cell carcinoma, and colorectal cancer [10, 51–53]. Recently, a study by Lu et al., which was the first to identify frequent miR-129-2 methylation in HCC, indicated the potential use of miR-129-2 methylation as a diagnostic marker for HCC [19]. Their results showed that 85% of stage I HCC patients could be identified based on their miR-129-2 methylation levels, with a cut-off value of 2.36 [19]. Furthermore, miR-129-2 methylation was not detected in plasma from patients with cirrhosis associated with hepatitis B or hepatitis C, which reflected the high specificity of this marker. Hence, miR-129-2 may be an early diagnostic marker for HCC, with the ability to distinguish HCC patients from cirrhosis patients and healthy individuals.

#### 3.2.7. miR-17-5p

miR-17-5p in the tissue has been confirmed in numerous studies to play an important role in carcinogenesis and invasion [54–56]. However, the value of miR-17-5p in HCC remained unknown. Zheng et al. conducted a study to measure the levels of serum miR-17-5p in HCC patients and focused on the predictive power of serum miR-17-5p for the prognosis of HCC [17]. Their results revealed that the levels of serum miR-17-5p were significantly lower in postoperative patients than in preoperative patients. In addition, compared with nonrelapsed patients, the levels of serum miR-17-5p in relapsed patients were elevated and nearly reached the levels of the preoperative patients. The authors also found significant associations of the levels of serum miR-17-5p with metastasis status and TNM stage. According to the above results, these investigators concluded that serum miR-17-5p can serve as a noninvasive biomarker for the prognostic prediction of HCC [17].

### 3.3. The Regulation of a Combination of Multiple Serum miRNAs in HCC

Results from Li et al.'s study demonstrated that although one particular miRNA in the serum may help distinguish different sample sets (i.e., miR-375/miR-10a for control and HBV-infected subjects, and miR-92a for control and HCV-infected subjects), a combination of multiple miRNAs may offer more specific testing [27].

### 3.4. A Plasma MicroRNA Panel

To identify the plasma miRNAs for diagnosing HBV-related HCC, Zhou et al. designed a clinical trial that included three phases [22]. In the discovery phase, 137 samples, each with 723 miRNAs, were screened with a microarray platform. Then, the 15 candidate miRNAs discovered via microarrays were selected for further testing by qRT-PCR in the training phase. Seven miRNAs (miR-122, miR-192, miR-21, miR-223, miR-26a, miR-27a, and miR-801) that were differentially expressed between the HCC and control groups (healthy, CHB, and cirrhosis) were further tested in an additional 305 participants. In the final validation phase, the parameters of the logistic model from the training phase were applied to an independent cohort of 390 samples for validating the diagnostic performance of the selected miRNA panel. Finally, the analysis of the results demonstrated that the miRNA panel had high accuracy in discriminating HCC from healthy tissue (AUC 0.941; 95% CI, 0.905 to 0.966; sensitivity 83.2%, specificity 93.9%), CHB (AUC 0.842; 95% CI, 0.792 to 0.883; sensitivity 79.1%, specificity 76.4%), and cirrhosis (AUC 0.884; 95% CI, 0.838 to 0.921; sensitivity 75.0%, specificity 91.1%) [22].

### 3.5. Combined Serum miR-15b and miR-130b

To investigate whether circulating miRNAs could outperform AFP for HCC detection, Liu et al. conducted a retrospective cohort study in two clinical centers [20]. In their study, combined miR-15b and miR-130b were demonstrated as a classifier for HCC detection. miR-15b may be involved in preventing replicative stress in response to mitogenic signaling [57]. This miRNA was recently shown to be overexpressed in HCC tumors [58]. On the other hand, miR-130b has been shown to be highly expressed in CD133+ tumor-initiating cells in HCC [59] and to directly target the well-known tumor suppressor RUNX3, suggesting the prominent oncogenic role of miR-130b in hepatocarcinogenesis [60]. In the results of the study by Liu et al., the detection sensitivity of combined miR-15b and miR-130b in a subgroup of HCCs with low AFP (<20 ng/mL) was 96.7%. Furthermore, the miRNA classifier could accurately identify 97.8% HCC cases from both tumor-node-metastasis stages I and II, whereas serum AFP (cut-off level at 20 ng/mL) could only detect 48.9% of the same cases [20]. Thus, the combined miR-15b and miR-130b classifier may be useful as a serum biomarker with clinical value for HCC screening.

## 4. Prospects

To improve the diagnosis of liver diseases, including HCC, researchers have made substantial efforts to develop noninvasive serum biomarkers. To avoid complications related to the heterogeneity of HCC patients, we discussed studies of HBV-related HCC patients in China only.

Over the past few years, significant alterations in the miRNA expression profiles between HCC and nontumor tissue have been demonstrated in many studies. Additional studies have shown the existence of a large amount of stable microRNAs in human serum/plasma and indicated that miRNAs may enter the circulation via active secretion from blood cells or tissues/cells. These studies laid the foundation for studying the role of serum/plasma microRNAs in the diagnosis and prognosis of HCC. In subsequent studies, many important discoveries have been made on the potential use of serum/plasma miRNAs as a novel noninvasive biomarker for HCC. Although considerable advances have been made, the reliability of these biomarkers is still debatable. Many questions remain to be addressed before certain miRNAs can be used as markers for HCC diagnosis, for example, the miRNAs that are deregulated in HCC patients involved in tumor development or the deregulation consequence of hepatocarcinogenesis, and why have previous studies often provided results that are contradictory [61]? Currently, there is no standard endogenous control that can be used in studies of circulating miRNA. Moreover, many previous studies have been limited by one or more of the following factors: poor study design, limited number of screened microRNAs, small sample size, failure to differentiate HCC from hepatitis B, and lack of independent validation. In addition, many miRNAs detected have yet to be validated across the heterogeneous population of HCC patients.

The use of serum miRNAs as biomarkers will likely improve the diagnosis of chronic liver diseases, including HCC. Furthermore, with a more detailed understanding of the source of serum miRNAs and the mechanisms that control the biogenesis of serum miRNAs, the use of particular miRNAs as noninvasive serum biomarkers for the diagnosis of some disorders may become the focus of future studies.

## Figures and Tables

**Table 1:** The detailed characteristics of included studies (Part 1).

Source	Number of patients	Age, Y	Gender (male : female)	Blood samples
2010 Li et al. [27]	**DS:** 30 HCC cases and 30 controls **TS:** 30 HCC cases and 30 controls **VS:** 55 HCC cases and 50 controls	37.52 ± 12.09 (range: 20–67)	74 : 86	All the blood samples were collected before any operation, chemotherapy, and/or radiation treatment

2011 Li et al. [25]	46 HCC cases and 20 HCs	<50, 18; *⩾*50, 28	33 : 13	Serum samples of HCC were obtained from patients prior to definitive therapy

2011 Gui et al. [26]	**DS:** 3 HCC cases and 2 HCs **VS:** 46 HCC cases, 23 CHB cases, and 24 HCs	HCC: 54 ± 9 CHB: 41 ± 9 HCs: 51 ± 6	HCC: 39 : 7 CHB: 17 : 6 HCs: 17 : 7	None of the enrolled HCC, ICC, and FNH patients had received any adjuvant therapy or other treatments before blood sampling

2011 Xu et al. [23]	101 HCC cases, 48 CHB cases, and 89 HCs	HCC: *⩽*50, 39; >50, 62 CHB: *⩽*50, 38 >50, 10 HCs: *⩽*50, 36 >50, 53.	HCC: 78 : 23 CHB: 31 : 17 HCs: 68 : 21	Preoperative and postoperative plasma samples were collected Postoperative plasma samples were obtained 10–30 days after surgery under the confirmation of no obvious recurrence by ultrasonography, CT, and/or MRI

2011 Qi et al. [24]	**DS:** 10 HCC cases and 10 HCs **VS:** 48 HCC cases, 48 CHB cases, and 24 HCs	HCC: 49 CHB: 45 HCs: 38	HCC: 55 : 15 CHB: 37 : 11 HCs: 24 : 10	Blood samples were taken at the time of initial consultation before definitive surgical intervention and/or adjuvant therapy

2012 Liu et al. [20]	57 HCC cases, 29 CHB cases, and 30 HCs	HCC: *⩾*60, 47; <60, 10 CHB: *⩾*60, 28; <60, 1 HCs: *⩾*60, 0 <60, 30	HCC: 49 : 8 CHB: 20 : 9 HCs: 22 : 8	Blood samples were collected before and 1 month after surgical resection

2012 Li et al. [21]	**DS:** 15 HCC cases and 15 HCs **VS:** 86 HCC cases, 30 CHB cases, and 45 HCs	HCC: 54 ± 11 CHB: 51 ± 13 HCs: 52 ± 16	HCC: 76 : 25 CHB: 23 : 7 HCs: 46 : 14	The preoperative serum samples were collected from 1 to 4 days before surgery, whereas the postoperative serum samples were collected from 8 to 359 days after surgery

2011 Zhou et al. [22]	**DS:** 57 HCC cases, 25 LC cases, 22 CHB cases, and 33 HCs **TS:** 204 HCC cases, 60 LC cases, 75 CHB cases, and 68 HCs **VS:** 196 HCC cases, 56 LC cases, 72 CHB cases, and 66 HCs	**TS:** HCC 53 ± 12 LC: 53 ± 13 CHB: 39 ± 13 HCs: 44 ± 11 **VT:** HCC 53 ± 12 LC: 50 ± 10 CHB: 39 ± 14 HCs: 45 ± 12	**TS:** HCC: 168 : 36 LC: 43 : 17 CHB: 48 : 27 HCs: 35 : 33 **VS:** HCC 166 : 30 LC: 40 : 16 CHB: 35 : 37 HCs: 43 : 23	No patients received surgery or chemotherapy or radiotherapy before blood sampling

2013 Lu et al. [19]	41 HCC cases, 8 LC cases, and 10 HCs	HCC: 57 ± 11.5 LC: 54.5 ± 6.6 HCs: 54.3 ± 14.5	HCC: 33 : 8 LC 5 : 3 HCs 5 : 5	No details

2013 Wei et al. [18]	No details	No details	No details	Blood samples were obtained from patients undergoing surgical HCC resection

2013 Zheng et al. [17]	96 HCC cases	<60, 40; *⩾*60, 52	HCC: 68 : 28	None of the enrolled HCC patients had received any adjuvant therapy or other treatments before blood sampling. The postoperative blood samples were collected one week after surgery and the relapsed blood samples were collected at the department of surgery

DS, discovery stage; TS, training set; VS, validation set; HCC, hepatocellular carcinoma; LC, liver cirrhosis; CHB, chronic hepatitis B; HCs, health controls.

**Table 2:** The detailed characteristics of included studies (Part 2).

Source	Healthy control	Internal control	Alterative microRNA	Conclusion
2010 Li et al. [27]	No details	plant miR-168	miR-25, miR-375, and let-7f	Serve as biomarkers for HCC diagnosis

2011 Li et al. [25]	No details	mmu-miR-295	miR-221	Provide significance for prognosis of HCC

2011 Gui et al. [26]	No details	U6 snRNAs	miR-885-5p	The detection and assessment of liver pathologies

2011 Xu et al. [23]	Healthy controls were randomly selected from a database consisting of 2500 individuals based on a physical examination	miR-181a and miR-181c	miR-21, miR-122, and miR-223	Serve as biomarkers for liver injury but not specifically for HCC

2011 Qi et al. [24]	No details	miR-16	miR-122	Serve as biomarkers for liver injury but not specifically for HCC

2012 Liu et al. [20]	Healthy controls are people who underwent routine physical examinations with no underlying liver diseases	miRNA concentrations were normalised to the total RNA input and expressed as the number of copies per nanogram of RNA	miR-15b and miR-130b	Serve as biomarkers for HCC diagnosis

2012 Li et al. [21]	Volunteers had not been diagnosed with any types of cancer previously based on self-report	U6 snRNAs	miR-18a	Serve as biomarkers for HCC diagnosis

2011 Zhou et al. [22]	No details	miR-1228	miR-122, miR-192, miR-21, miR-223, miR-26a, miR-27a, and miR-801	Serve as biomarkers for early-stage HCC diagnosis

2013 Lu et al. [19]	No details	U6 snRNAs	miR-129-2	Serve as biomarkers for early-stage HCC diagnosis

2013 Wei et al. [18]	No details	U6 snRNAs	miR-132	miR-132 may be a promising biochemical marker and may have therapeutic applications in HBV-related HCC

2013 Zheng et al. [17]	No details	U6 snRNAs	miR-17-5p	Serve as a noninvasive biomarker for the prognostic prediction of HCC patients.

snRNAs, small nuclear RNAs; HCC, hepatocellular carcinoma.
